# Perspective: Assessing Tolerance to Nondigestible Carbohydrate Consumption

**DOI:** 10.1093/advances/nmac091

**Published:** 2022-08-30

**Authors:** Hannah D Holscher, Bruno P Chumpitazi, Wendy J Dahl, George C Fahey, DeAnn J Liska, Joanne L Slavin, Kristin Verbeke

**Affiliations:** Department of Food Science and Human Nutrition, University of Illinois, Urbana, IL, USA; Department of Pediatrics, Baylor College of Medicine, Houston, TX, USA; Children's Nutrition Research Center, United States Department of Agriculture, Houston, TX, USA; Department of Food Science and Human Nutrition, Institute of Food and Agricultural Sciences, University of Florida, Gainesville, FL, USA; Department of Animal Sciences, University of Illinois, Urbana, IL USA; Independent Consultant, Ridgefield, WA, USA; Department of Food Science and Nutrition, University of Minnesota, Twin Cities, MN USA; Translational Research in Gastrointestinal Disorders, KU Leuven, Targid, Leuven, Belgium; and Leuven Food Science and Nutrition Research Centre, Leuven, Belgium

**Keywords:** dietary fiber, oligosaccharides, low-digestible carbohydrates, laxation, gastrointestinal intolerance

## Abstract

Human intestinal enzymes do not hydrolyze nondigestible carbohydrates (NDCs), and thus, they are not digested and absorbed in the small intestine. Instead, NDCs are partially to completely fermented by the intestinal microbiota. Select NDCs are associated with health benefits such as laxation and lowering of blood cholesterol and glucose. NDCs provide functional attributes to processed foods, including sugar or fat replacers, thickening agents, and bulking agents. Additionally, NDCs are incorporated into processed foods to increase their fiber content. Although consumption of NDCs can benefit health and contribute functional characteristics to foods, they can cause gastrointestinal symptoms, such as flatulence and bloating. As gastrointestinal symptoms negatively affect consumer well-being and their acceptance of foods containing NDC ingredients, it is crucial to consider tolerance when designing food products and testing their physiological health benefits in clinical trials. This perspective provides recommendations for the approach to assess gastrointestinal tolerance to NDCs, with a focus on study design, population criteria, intervention, comparator, and outcome. Special issues related to studies in children and implications for stakeholders are also discussed. It is recommended that the evaluation of gastrointestinal tolerance to NDCs be conducted in randomized, blinded, controlled crossover studies using standard gastrointestinal questionnaires, with attention to study participant background diets, health status, lifestyle, and medications.

## Teaser

**Statement of significance**: This perspective provides recommendations for research approaches to assess gastrointestinal tolerance to nondigestible carbohydrates by providing a roadmap for the study design, population criteria, intervention, comparator, and outcomes.

## Introduction

Human intestinal enzymes do not hydrolyze nondigestible carbohydrates (NDCs), and thus, they are not absorbed in the small intestine (1). These carbohydrates may be partially to completely fermented by the intestinal microbiota (2). Many NDCs are dietary fibers, defined by the FDA as “nondigestible soluble and insoluble carbohydrate, and lignin that are intrinsic and intact in plants, and isolated or synthetic nondigestible carbohydrates determined by the FDA to have physiological effects that are beneficial to human health” (3). There are many health benefits associated with NDC consumption, particularly those considered to be dietary fibers, including lowering blood glucose and cholesterol concentrations, increasing intestinal calcium absorption, reducing energy intake, and improving laxation (4). In addition to health benefits, NDCs provide functional attributes to processed foods, such as sugar or fat replacers, thickening agents, and bulking agents (5). NDC use in processed foods is commonplace because of their health benefits and functional properties.

**FIGURE 1:**
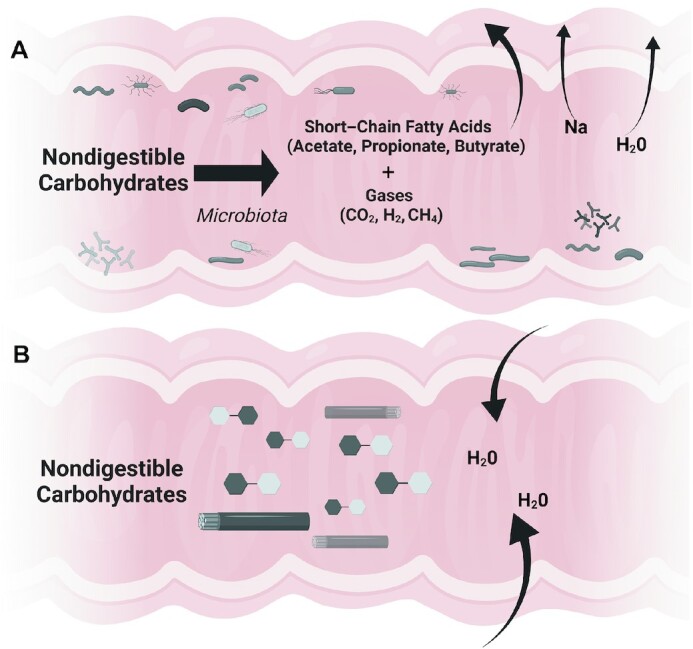
Microbial fermentation of NDCs results in short-chain fatty acids (SCFA) and gas production, which contributes to sodium and water absorption (A). The osmotic effects related to the presence of low molecular weight NDCs within the intestine draw water into the lumen (B). Created with BioRender.com. NDC, nondigestible carbohydrate.

Many health benefits of consuming NDCs relate to their nondigestible properties and role as fermentable substrates for the intestinal microbiota. Microbial fermentation of NDCs produces short-chain fatty acids (SCFAs), which in addition to their energy provision, increase sodium and water absorption within the intestine (6), **Figure 1**A. However, NDC fermentation also produces gases that can induce gastrointestinal effects, such as bloating, flatulence, and abdominal discomfort (7, 8). Additionally, the osmotic effects related to the presence of low molecular weight NDCs within the intestine (Figure 1B) can affect stool form and consistency, and, rarely, excessive intake may result in diarrhea. Although responses to NDC intake may be mild and transitory and not considered safety concerns or serious adverse events in clinical trials, documentation and reporting of gastrointestinal tolerance outcomes are needed for interpreting the science and informing recommendations. Indeed, following the completion of clinical trials that establish the safety of novel ingredients, including NDCs, attention to gastrointestinal effects is an area of key concern. As gastrointestinal symptoms can negatively affect consumer acceptance of foods containing NDC ingredients, it is vital to consider tolerance when designing food products and testing physiological health benefits related to their consumption. In this perspective, we provide a roadmap for the approach needed to assess gastrointestinal tolerance to NDCs as a primary outcome in interventional trials, discuss special considerations for studies in infants and children, and reflect on implications for stakeholders from a US perspective. A summary of key conclusions is shown in **Box 1**.

Box 1. Main recommendations for assessing tolerance to nondigestible carbohydrates

- Randomized, blinded, controlled crossover trials are recommended.
- For acute tolerance studies with 1-time challenges, monitor symptoms for a minimum of 0–48 h followed by a  ≥3-d washout before the next test.
- For acclimation studies, assess tolerance outcomes over ≥14 d, followed by a washout period of similar length.
- Capture baseline symptoms before an acute challenge or during a lead-in before an acclimation study.
- Fully report the NDC characterization and how the NDC is consumed (e.g., purity of NDC, food form and dose,).
- Negative comparators should match the food product but contain no NDC ingredient.
- If used, the dose of positive controls should be similar to the treatment dose.
- Life stage, health, dietary, lifestyle, and medication factors should be considered when establishing inclusion/exclusion criteria.
- Assess subjective participant-reported symptoms via standard gastrointestinal questionnaires as the primary tolerance measures.
- Objective outcome parameters can complement gastrointestinal questionnaires but should not replace them.

## Methods

A scientific symposium was organized on Considerations for Designing a Protocol to Evaluate Tolerance of NDCs at the December 2020 12th Annual Vahouny Fiber Conference. Before the meeting, speakers developed presentations on core issues related to designing studies to assess tolerance, including considerations for the population, intervention, comparator, and outcomes. During the scientific symposium, the presentations were followed by a panel discussion on issues presented by stakeholders. After the meeting, HDH, BPC, WJD, DJL, and KV wrote sections of this perspective, which HDH compiled into a draft report. All authors critically reviewed, edited, and agreed upon this document.

### Study design

Significant interindividual variation in gastrointestinal symptoms is reported in healthy cohorts (9); thus, a randomized, double-blind, controlled, crossover design is recommended when tolerance to an NDC is the primary outcome. When possible, a placebo control is recommended. However, if the NDC is tested as a food ingredient compared with a supplement, a control food (as opposed to a placebo) may be necessary as it may not be feasible to achieve double blinding given perceptible differences between the intervention and the control items.

The intervention and washout period durations will depend on whether the research question relates to acute outcomes following a 1-time challenge or adaptation to daily consumption. Symptoms from an acute or first consumption event generally occur within the first 12–24 h and begin to subside within 48 h (10). Whole-gut transit time for healthy adults generally ranges from 1 to 3 d (11). Thus, for acute tolerance studies with 1-time challenges, symptoms should be monitored from 0 to 48 h minimum, extending to 72 h if the return to baseline is also of interest. Study participants should undergo at least a 3-d washout before the next test. However, a 6-d washout may be indicated to accommodate individuals with slower transit times (11–13). For chronic studies, colonic gas production following NDC consumption returns to baseline 14 d after initiating consumption (14). Thus, tolerance outcomes should be assessed over at least 14 d for acclimation studies, followed by a washout period of a similar length. The first day or more of tolerance data during an intervention period may reflect the baseline or lead-in diet and associated microbiota activity. Thus, data from week 2 and beyond may better reflect the responses to chronic NDC consumption. However, such short-term studies still may not reflect long-term acclimation. Evidence is mounting that shifts in the microbiota composition and, possibly, associated tolerance to NDCs, may require longer-term intake (15).

Other special considerations include lead-in periods. The NDC being evaluated should be eliminated from the diet for ≥6 d before the study intervention period. To limit confounding, foods and beverages containing the NDC under investigation should be restricted. However, given the ubiquitous nature of NDCs in foods, it is increasingly challenging to control the background diet during interventional studies.

We also encourage assessment of gastrointestinal tolerance to NDCs as secondary outcomes in clinical trials. For studies that aim to demonstrate a physiological benefit of an NDC so that it may be considered a dietary fiber by the FDA, the FDA provides guidance for considerations relevant to study design, comparators, analysis, and interpretation (16). Specific to assessing acute tolerance outcomes, we recommend that symptoms be monitored from 0 to 48 h minimum. For acclimation outcomes, tolerance symptoms assessment should be assessed over at least 14 d. Collecting the same tolerance outcome measures at baseline, i.e., before the intervention, is optimal to determine whether a change in gastrointestinal symptoms has occurred and delineate between responders and nonresponders. This is particularly critical in parallel-design studies, as symptom frequency and intensity may differ between groups at baseline.

### Intervention and control

#### Characterization of nondigestible carbohydrates

NDCs comprise a wide range of naturally occurring and synthetic or modified food ingredients. Their chemical structures predict functionality (e.g., viscosity and fermentability), affecting the physiological responses related to tolerance (17). The foremost consideration when planning tolerance assessment studies is fully characterizing the NDC of interest. For transparent reporting, to provide sufficient information for study replication and to compare results across tolerance studies, details regarding the composition of the NDC, including chemical structure (molecular weight, composite sugars, and linkages), particle size, and its functionality, such as viscosity and fermentability, should be appropriately analyzed and reported. Additionally, it is imperative that the quantity of the NDC administered and the fraction of the full ingredient amount demonstrated to be nondigestible be reported. Selecting NDC suppliers who will provide this critical information as often as possible and considering independent analysis to supplement this information further will provide additional vigor to the study.

#### Amount of NDC for testing

The current “fiber gap,” the difference between actual fiber consumption and the recommended fiber consumption, averages ∼15 g/d for Americans (18). This gap could be closed by adding NDC ingredients (specifically those considered dietary fibers) to various processed foods (19). However, for tolerance testing, the appropriate level per serving first needs to be considered. For food product labeling purposes in the United States, the daily value (DV) for fiber set by the US FDA is 28 g/d; 10% of the DV (minimum 2.8 g/serving) may be labeled as a “good source” of fiber, and 20% of DV (minimum 5.6 g/serving) as an “excellent source” (20). Thus, tolerance studies on the new NDC ingredients considered dietary fibers and used in food products on the market should consider foods formulated at these levels, as well as a higher daily amount, perhaps 10–15 g/d, to account for multiple servings/d. A 2-g serving amount is unlikely to elicit measurable tolerance issues in healthy adults, so a minimum level of 3 g, to reflect a good source, is recommended. Testing levels of 3 g and 6 g per serving would be appropriate to reflect putative label claims. Regarding testing levels for markets outside the United States, consider the regulatory agency determinations in those markets.

In addition to testing levels of an NDC on a per-serving basis, a harmonized daily intake level for tolerance testing is recommended. This level would be appropriate to evaluate tolerance as a secondary outcome of an efficacy study. Viscous fibers have been frequently studied for their efficacy in improving cardiometabolic outcomes such as serum cholesterol concentrations and glycemic control. Meta-analyses on psyllium and other viscous NDCs have provided information on an appropriate daily intake level for testing. For instance, consuming 7–15 g/d psyllium demonstrates efficacy for cholesterol lowering as adjunctive therapy with statins (21). A median dose of 13 g/d of viscous fiber reduces HbA_1c_, fasting blood glucose concentrations, and insulin resistance (22). These studies demonstrate that a daily intake of 15 g/d of viscous NDCs may be appropriate for testing the efficacy of cardiometabolic outcomes and tolerance. For nonviscous NDCs, the health outcome tested in efficacy studies is often laxation—a meaningful change in transit time or stool frequency in a healthy population. Less fermentable NDCs predictably increase fecal weight and decrease transit time when transit is >2 d (23). In constipation studies, 5–15 g/d of NDCs have induced symptom relief (24). In healthy adults, modeling suggests 10 to 18 g/d of highly fermentable short-chain (DP <10) β-fructans increase stool frequency (25). Thus, a daily intake of ∼15 g/d of NDCs also may be appropriate for tolerance testing when the primary outcome is laxation in healthy adults.

For the regulatory labeling reasons described above, the total daily dose of 15 g of NDCs should be divided into 3 to 5 servings/d “good source” or “excellent source.” However, such a dose distribution may attenuate the gastrointestinal symptom response to NDCs. Specifically, consuming 3 to 5 g of added NDCs per serving may elicit milder gastrointestinal symptoms than a single dose of 15 g. However, the distribution of NDC-containing foods throughout the day is likely more reflective of how these foods would be consumed. Timing of intake also may impact tolerance outcomes. For example, consuming the NDC in the evening hours may result in less symptom reporting than intake earlier in the day, given that symptoms often manifest within the first 12 h. Ultimately, if the total dose is divided throughout the day, this information should be appropriately reported.

Tolerance differs with the type and amount of NDC consumed and is affected by the highly individualized response, as discussed in the Study Population section below. This is partly due to the speed and extent of fermentation by the intestinal microbiota, especially when assessing bloating and flatulence. For example, inulin, a highly fermentable fiber, is considered acceptable or well tolerated in the generally healthy population at daily intakes of up to 15 g (26). In contrast, the acceptable daily intake of polydextrose, a slowly fermented substrate (27), may exceed 40 g (26). Reported gastrointestinal symptoms and symptom intensity typically increase with consumption amounts for a highly fermentable NDC (28). In contrast, NDCs entirely resistant to fermentation may not exhibit a different effect with higher intakes (29). Harmonizing testing levels per serving and daily intakes will allow comparisons among NDC ingredients based on consumer tolerance. Dose–response studies will inform how NDC intake levels correspond to the gastrointestinal symptoms reported.

#### Food form

Another consideration for tolerance testing is the food form of choice, such as beverage or solid food. The study product should be considered within the context of the complete diet, and the formulation of study products needs to be selected with consideration of the NDCs already present in the chosen vehicle (e.g., fiber content inherent within a whole-grain bar before adding NDCs). Food form may influence gastric emptying (30), affecting the speed at which fiber arrives in the colon, is fermented and potentially produces symptoms. Solid food remains in the stomach longer than liquids, and high-calorie liquids longer than low-calorie liquids or water. Thus, tolerance response to a beverage may differ from that to solid food. Further, food processing, such as heating and cooling cycles, may influence tolerance outcomes as the structure, molecular weight, and bioavailability of total NDC content may be impacted by processing (31). Ultimately, the intervention and the control items should be as similar as possible, and detailed information about the study food, including key nutrient components (i.e., energy, fat, protein, carbohydrate, and NDC), processing, and storage, should be reported. See Weaver et al. for detailed guidelines regarding study foods when conducting human nutrition randomized controlled trials (32).

#### Comparator

When considering efficacy studies for demonstrating that an NDC is a fiber, the FDA states that an “appropriate” control for the primary outcome is needed (16). Although tolerance is not considered a beneficial physiological outcome per the FDA, improved laxation is. Ultimately, the standard choice for a comparator is a negative control—a food or beverage that closely matches the test product but contains no NDC ingredients. However, a positive control is also recommended because the tolerance response is individualized, and the outcome assessed is subjective. A positive control should be an NDC with a documented efficacy endpoint of interest when tolerance is a secondary outcome. Alternatively, an NDC with documented tolerance data is an appropriate positive control (26). Ultimately, the positive control should be a well-described NDC added at a similar level to the ingredient of interest. As research advances, the appropriate choice of a positive control may well be based on the speed and extent of fermentation, characteristics that may contribute to gastrointestinal symptom response, and, thus, tolerance. Characterizing the fermentability by in vitro testing may guide the choice of the comparator (33).

#### Dietary control

Recommendations for acute (single meal) tolerance studies include standard practices such as those outlined for glycemic response studies, i.e., a 10-h overnight fast, set time to consume study food, and served with a standardized amount of water (34), as well as a consistent symptom assessment period (i.e., 0 to 48-h minimum). For longer studies, the time of day of consumption and whether or not the study food is consumed with meals may affect tolerance experiences and symptom reporting and should also be standardized. Compliance to study food intake is a challenge with longer trials and is difficult to document well, given there is no objective biological marker for NDC intake.

Monitoring the background diet is essential. We propose 2 acceptable approaches—dietary control (i.e., complete feeding) or having participants consume their usual diet. Tight dietary control using a full-feeding protocol may ensure high sensitivity for tolerance assessment but likely also affect the intestinal microbiota and through fermentation, gas production and gastrointestinal symptoms (35). A lead-in period of 3 to 6 d on the controlled study diet is recommended to account for the amount of time needed for food residues to pass through the whole gut (12, 13); however, longer lead-in periods of 2 to 6 wk may be necessary to ensure adequate washouts of restricted foods or supplements [i.e., probiotics, prebiotics, and synbiotics (36, 37)]. Alternatively, tolerance to an NDC may be assessed with study participants consuming their usual diets, similar to how the food product would be included in a consumer's dietary pattern. For acute studies, replication of the participants’ diets for the 24–72 h before test product consumption is encouraged (38). Regardless of the approach utilized to control the diet, detailed information on the study participants’ dietary intake should be collected, including their background fiber intake. Recording dietary intake is necessary to facilitate comparisons within and among NDC intervention studies.

### Study populations

The selection of the study population is an important factor in clinical studies on gastrointestinal tolerance of NDCs intended for the general population. Studies conducted for safety purposes (e.g., laxation threshold determination, generally recognized as safe documentation) frequently involve very high levels of the test NDC and include participants who are usually younger and very healthy. These studies provide valuable information on the safety profile of a substance for regulatory filings; however, they represent only a subgroup of the general population and may not fully reflect what will occur with the food in the market. This perspective aims to guide the criteria for inclusion of participants who would represent individuals who consume the NDC within foods available on the market, i.e., the general population. **Table 1** provides a summary of the study population suggestions.

**TABLE 1:** General population selection for studies on gastrointestinal tolerance^1^

Criteria	Comments
Male or female	Exclude pregnant and lactating people, and those with irregular bowel habits associated with their menstrual cycle, or plan test days to occur at similar times of their menstrual cycle for acute studies.
Age <18 vs. 18 to 65 y (inclusive)	The age range should reflect the age targeted for the NDC use and could be extended up to 75 y. For children or adolescents, a separate study should be conducted. Validated pediatric-friendly measures should be used in children.
Ethnicity, race	As inclusive as possible to include those who will consume the product and allow for generalizability to the population.
BMI ≥18.5 to <40	There is no specific requirement unless the ingredient or product is targeted for individuals with a particular body mass index range. Inclusion criteria may include those up to 40 kg/m^2^ but should consider criteria for metabolically healthy obesity (i.e., no components of the metabolic syndrome, except high waist circumference), comorbidities, and medications.
Standard diet	Participant typical diet should contain low-to-average fiber consistent with the population. Participants should maintain habitual dietary patterns, avoid foods that cause gastrointestinal distress and high-fiber foods during the run-in and study periods. Exclude individuals on extreme diets (e.g., vegan, gluten-free, paleo, weight-loss, etc.).
Allergies and food sensitivities	Exclude individuals with allergies to any of the test product components. Reported sensitivities to NDCs should be noted for possible sensitivity analysis or subgroup assessments.
Generally healthy	Exclude individuals with a history or presence of relevant endocrine, cardiovascular, pulmonary, biliary, renal, hepatic, pancreatic, or neurologic disorders. May include individuals at-risk or with certain stable chronic health conditions as long as the disease and treatment would not interfere with the test substance's evaluation. Exclude those with trauma or surgical event within 2 mo or having a cancer diagnosis within 2 y, except for nonmelanoma skin cancer.
Gastrointestinal health	Exclude individuals with diagnosed gastrointestinal disease or conditions, including inflammatory bowel disease, celiac disease, and disorders of the gut–brain interaction such as clinically diagnosed irritable bowel syndrome, as well as individuals with presence or history of gastrointestinal cancer. Exclude individuals with recent history (within 6 wk) of constipation, diarrhea, or other gastrointestinal illness such as nausea or vomiting. For some studies, depending on the participant number and product population target, it may be reasonable to exclude individuals with known irregular bowel habits. Bowel habits and gastrointestinal symptoms should be documented during an appropriate run-in or baseline period.
Psychiatric conditions	Exclude individuals with diagnosed psychiatric disorders that impede their ability to differentiate and communicate symptoms.
Medications	Either exclude individuals on prescribed medications or include those on stable medications, except for medications that have gastrointestinal side effects or affect carbohydrate digestion (e.g., α-glucosidase inhibitors, antidiarrheals, and laxatives). Although most past studies have excluded individuals on cholesterol-lowering medications, proton-pump inhibitors, and metformin, due to the increased use of these medications in the general population, inclusion could be considered, as long as participants have been on the medication for >6 mo, with regular use and not exhibiting gastrointestinal side effects.
Antibiotics	Exclude individuals with recent (within 6 wk) antibiotic use. Exclude from the analysis those who took antibiotics for a medical condition during the study.
Supplements	Willing to avoid fiber, prebiotic, and probiotic supplements, as well as supplements that affect carbohydrate metabolism or gastrointestinal function (e.g., antidiarrheals, laxatives, etc.). Stop usage ≥2 wk before study initiation and during the entirety of the study.
Physical activity	Willing to maintain habitual physical activity patterns. Exclude individuals with extreme physical activity patterns (e.g., marathon training).
Smoking	Exclude heavy smokers (>19 cigarettes/d) and document number of cigarettes per day in smokers enrolled in the study.
Alcohol and unregistered drugs	Exclude those that report alcohol or unregistered drug abuse. Alcohol abuse is defined as >14 drinks per wk (1 drink = 0.6 fluid oz or 14 g of pure alcohol).

1 NDC, nondigestible carbohydrate; vs., versus.

The general population includes many people at risk for, or with, ≥1 chronic diseases. The CDC indicated that 1 in 4 American adults has 1 chronic disease, and 1 in 6 has >1 chronic disease (39). Similar to studies on NDCs for identifying a fiber benefit and acceptance as a dietary fiber by the FDA, studies that aim for their outcomes to be translatable to the general population should include generally healthy people as well as individuals at risk of chronic disease (e.g., elevated LDL cholesterol concentrations, abnormal glucose tolerance test) (16).

The prevalence of gastrointestinal symptoms in the population also should be considered. Symptoms of disorders of gut–brain interaction, formally known as functional gastrointestinal disorders (40, 41), which include irritable bowel syndrome, functional dyspepsia, and functional constipation, are experienced by ≤40% of the population at some point in their life (42). The most common disorder of gut–brain interactions is irritable bowel syndrome, with a global prevalence of 1 in 10 people. As many as 16% of the US population have irritable bowel syndrome (43). Those with a medical diagnosis of a disorder of gut–brain interactions should be excluded. Some individuals may identify themselves as having a disorder of gut–brain interactions but have not been diagnosed by a physician. These individuals could be excluded due to the instability of their symptoms; however, given the prevalence of gastrointestinal symptoms in the general population, this may limit the available study population. Therefore, excluding those experiencing chronic symptoms is a reasonable approach. However, due to the commonality of symptoms, care should be taken to document each individual's gastrointestinal symptom history. Statistical analysis can *a priori* include the option to conduct a sensitivity assessment that excludes these individuals. Those with gastrointestinal diseases, such as inflammatory bowel disease and celiac disease, should be excluded, as well as those with a presence or history of gastrointestinal-related cancer or surgeries (e.g., bariatric surgery).

Demographics and general factors, such as age and BMI (in kg/m^2^), can be broad to represent the general adult population. The intended population that will consume the product that contains the NDC is the best determinant for selecting the age range. Tolerance assessments in children and adolescents should be conducted in separate studies and can be done in parallel with an adult study (see the Special Considerations for Children section below). For adults, males and females ≥18 y old should be included. Currently, the cutoff for defining a person in the category of older adults is 65 y. In 2019, approximately 17% of the US population was >65 y old, with an overall national median age of 38.4 y (44). Older adults have changes in many aspects of gastrointestinal function (45). Therefore, many studies include adults aged ≥18 and ≤65 y. However, due to increased life expectancy and people maintaining health longer, as well as the anticipated increase in older adults over the next few decades (expected to reach >23% by 2060), the age range should include adults aged ≤75 y to better reflect the general healthy adult population. Specific to exclusionary criteria based on BMI, a general recommendation is to include individuals with BMI between 18.5 and 39.9, thereby excluding anyone with class 3 (severe) obesity and above. Alternatively, an approach to include those with metabolically healthy obesity (those without any components of the metabolic syndrome, except for high waist circumference) (46) can be undertaken. No requirements for race or ethnicity are indicated. The study should be conducted in a location that will recruit a broad population for racial, ethnic, and age diversity. Stratification can be considered to ensure groups are balanced on individual factors.

Safety studies on new products and NDCs are often conducted in males only. In females, disorders of gut–brain interaction such as irritable bowel syndrome are more prevalent, and there is a correlation between ovarian hormone cycles and irritable bowel syndrome symptomatology (47, 48). Some females without diagnoses of a disorder of gut–brain interactions also may report irregular bowel habits during their menstrual cycle, and there are differences in gastrointestinal motility in females compared to males (49). Females should be included in studies intended to represent the general population; however, females noting irregular bowel habits and gastrointestinal symptoms during portions of their menstrual cycles can be excluded or, depending on study design, menstrual cycle timing can be documented and accounted for when planning study participation and testing dates.

Diet and lifestyle factors also are important criteria for selecting participants to represent the general population. Many diet and lifestyle factors affect gastrointestinal function and vary among the population (26). Participants should have dietary habits that represent the general population (e.g., not on a strict weight loss plan or following specific strict practices), not participate in extreme physical activity practices (e.g., marathon training), and have average fiber intakes. The average intake for the US population aged ≥20 y is ∼16.9 g of fiber (18.4 for males and 15.5 for females) (50). Background diet and physical activity should remain consistent throughout the study.

Participants should be counseled to avoid introducing new foods or making significant lifestyle changes, and changes during holidays should be avoided when possible, particularly those that involve the introduction of changes in diet and lifestyle routines. Participants who disclose alcohol or drug abuse should be excluded. Smoking affects gastrointestinal integrity and is associated with inflammatory bowel disease (51). Individuals who are heavy smokers, generally defined as >19 cigarettes/d, have been reported to have a relatively poorer diet, higher alcohol intake, and lower physical activity levels (52). We recommend excluding individuals who use tobacco products. However, if such individuals are included, enrollment should be limited to those with low- or moderate-level smoking behavior (<10 tobacco products/d).

Finally, prescribed and over-the-counter drug and supplement use should be queried. Some medications elicit gastrointestinal-related adverse events. For example, chronic use of nonsteroidal anti-inflammatory drugs is associated with many digestive and gastrointestinal side effects, and statins, proton pump inhibitors, fibrates, metformin, β-blockers, and iron supplements can cause constipation or diarrhea (53). Therefore, it is preferable for individuals on medications for which gastrointestinal side effects are noted to be excluded from tolerance studies. However, given the prevalence of the use of these medications, such individuals may be included to reflect the general population, as long as medication use is recorded and the participants have been on stable doses for ≥6 mo with no recent history of gastrointestinal-related adverse events from the drug. Individuals actively taking prescribed antibiotics should be excluded. A washout period of at least 6-wk following completion of an antibiotic prescription is recommended before enrollment to avoid instances of late-onset antibiotic-associated diarrhea (54, 55). Individuals using fiber, prebiotic, or synbiotic supplements should be excluded unless willing to discontinue. If these individuals discontinue consuming these products, a washout period of ≥6 d should be conducted (11–13). Similarly, those consuming certain probiotic or synbiotic supplements or foods with added probiotics that affect gastrointestinal tolerance or stool habits should be excluded. Alternatively, participants may cease consumption of the probiotics before study enrollment. In those instances, care should be taken to ensure an adequate washout period, which is strain specific (37). For example, consuming fermented milk with the probiotic strain *Bifidobacterium animalis* DN-173 010 reduced orofecal transit time in elderly individuals up to 6 wk after consumption of the product was stopped (36). Consumption of fermented foods is allowable as most fermented foods sold commercially are not probiotic fermented foods (56). As with all dietary intake, consumption of fermented foods should be documented within the dietary records.

Ultimately, even with similar inclusion/exclusion criteria, studies may end up with differing participant profiles due to the study's location and the available population. Therefore, it is vital that researchers fully report inclusion and exclusion criteria and the demographics of the study population.

### Sample size and analysis

The number of participants in published tolerance studies has ranged from <10 to >100, with the most common number ∼20–50 participants (26). A power calculation should be conducted *a priori* to determine the number of participants needed to detect a statistically significant difference between the control and the intervention (i.e., the product containing the NDC). Analyses should include adjustments for multiple comparisons where appropriate (16). Examples of power calculations based on total gastrointestinal symptoms are available in the published literature (57–59). However, data often are not available for the specific NDC under study. Thus, data from similar NDCs should be utilized to determine the clinically relevant minimum effect. In addition, it has been shown that for some NDCs, some people react while others do not, and the percentage of “responders” provides clinically relevant information on the effect of consuming the NDC. Examples for reporting responders are also available in the published literature (58,59). Therefore, the number of participants should be determined based on the power analysis to detect a statistically significant difference in total scores and reporting of the percentage of responders and the anticipated attrition rate. Overall, broad inclusion criteria may require more participants to ensure full representation for generalizability to the general population, with a minimum of 50–60 participants per arm or total for a crossover recommended.

### Outcomes

Digestive tolerance can be described as the interaction between the digestive and fermentative processes within the gastrointestinal tract and the subjective responses individuals have to these normal physiological processes. Several outcomes can be used to assess digestive tolerance to NDCs, and the type of outcome will depend on the study design. Small-scale or mechanistic studies might include sample collection for analysis of specific markers (i.e., objective outcomes), whereas participant-reported outcomes such as questionnaires (i.e., subjective outcomes) might be the primary outcome in large population-based studies or observational studies.

#### Participant-reported outcomes

As digestive tolerance is related to the individual perceptions of the digestive and fermentative process occurring within the gastrointestinal tract, questionnaires are the most appropriate tool to assess gastrointestinal tolerance. Symptoms assessed often include gas/bloating, nausea/vomiting, flatulence, abdominal distension, abdominal pain/cramping, reflux, burping, and borborygmus. We recommend assessing symptoms daily over the first 48 to 72 h for acute studies and during the baseline and final week of chronic trials. See **Supplemental Figure 1** for an example of a standard daily gastrointestinal tolerance log, including stool consistency ratings using the Bristol Stool Chart (**Supplemental Figure 2**). Stool habits are relevant to the tolerance of NDC consumption. The Bristol Stool Chart (70–72) has become the gold standard for assessing stool consistency in epidemiological and clinical studies (73). Using this chart, participants can classify their stools into 7 categories based on their appearance, going from hard lumps (type 1) to entirely watery stools (type 7). Furthermore, the Bristol Stool Chart score correlates reasonably well with measurements of whole-gut transit times, at least in adults with widely varying transit times, and can pick up pharmacologically induced changes in transit (71).

When symptoms are evaluated over a longer period, e.g., the previous week, it is recommended to score the severity and the frequency of the symptoms. Scoring can be performed using visual analog scores, although Likert-type scales often are preferred because processing is easier. An example of a 4-point Likert-type scale for severity is 1 = none, 2 = mild, 3 = moderate, and 4 = severe, and for frequency 1 = less than usual, 2 = as much as usual, 3 = somewhat more than usual, 4 = much more than usual. An example of a standard weekly questionnaire (**Supplemental Figure 3**) that has been utilized in fiber tolerance studies in healthy adults was published by Maki et al. (57) and subsequently utilized by others in healthy adults to study the effects of NDC consumption (60, 61). Alternatively, the Gastrointestinal Symptom Rating Scale (GSRS) is an example of a validated tool that queries symptoms over the past week; however, it should be noted that this tool was developed as a disease-specific instrument (62, 63). Normal values for the general population are available (64). The GSRS has been used to assess symptoms in healthy adults consuming NDC (65–67).

In addition to gastrointestinal symptoms, carbohydrate malabsorption may lead to extra-gastrointestinal symptoms such as headache, tiredness, or reduced concentration. In a study of 2042 patients with disorders of gut–brain interaction, up to 50% reported tiredness after a challenge with fructose or lactose (68). Whether these symptoms also occur in healthy participants after a challenge with NDCs has not been investigated.

Questionnaires are generally processed into a composite score that is the sum of the scores of the individual symptoms, and the resulting value is subjected to statistical analysis. However, especially in large populations, statistically significant increases in symptom scores do not necessarily equate with clinically relevant effects. Relevant effects could be defined as ≥1 symptom scored as moderate or severe or by specifying a threshold in the allowable increase in the composite score.

Finally, it is recommended that gastrointestinal questionnaires be completed in the same setting throughout a study. Bovenschen et al. reported a 37% difference in the score when questionnaires were completed by the participants at home compared with during an interview with the investigator (69).

#### Objective outcomes

Objective outcomes, i.e., parameters that can be measured and quantified, can complement gastrointestinal questionnaires and may be helpful for better understanding of intolerance symptoms. However, such outcomes do not facilitate measurement of tolerance and, therefore, do not replace the gastrointestinal questionnaires.

Abdominal discomfort after NDC intake is thought to be caused by excessive gas production in the colon. The hydrogen breath test can estimate the amount of gas based on the principle that human enzymes do not produce hydrogen. Hydrogen in humans originates from microbial fermentation of carbohydrates and is partially exhaled in breath or eliminated via flatus (74). The hydrogen breath test was developed as a qualitative test to detect malabsorption of digestible carbohydrates such as lactose and fructose. As NDCs are indigestible and reach the large intestine, a rise in hydrogen in breath is expected depending on its fermentability. However, the fraction of hydrogen that appears in breath depends on the production rate and is not proportional to the total amount produced in the colon (75). Therefore, breath hydrogen only approximates but does not accurately reflect total hydrogen production in the gastrointestinal tract. Correlations between hydrogen in breath and symptoms have been evaluated mainly in the context of sugar malabsorption. In a retrospective analysis of 1051 lactose malabsorption tests, discomfort in patients diagnosed with lactase deficiency was associated with significantly higher hydrogen in the breath (76).

Stool parameters can be measured directly in addition to participant-reported outcomes, such as the Bristol Stool Scale. Stool output can also be measured, and stool consistency can be quantified as the percentage of dry weight obtained after drying the fecal samples. Yet these measurements require the collection of stools and manipulation by laboratory staff, which is cumbersome and unpleasant. Transit can be estimated from the position of radio-opaque markers upon X-ray. Participants must swallow radiologically distinguishable markers on separate days, after which an abdominal X-ray shows the marker positions and allows for transit time calculations (77). Alternatively, mean gastrointestinal transit time can be calculated following radio-opaque marker intake by tracking defecation times and marker counts from X-rays of complete stool collections (12) or by a single stool method (78). Transit time can also be noninvasively assessed using marker dyes (11, 79).

#### Exploratory outcomes

Some exploratory outcomes might be useful to better understand differences in tolerance to different NDCs.

##### Microbiota composition

Fecal microbiota analysis may explain the differences in gas production after consuming different NDCs. The total amount of hydrogen in the intestines is the net result of hydrogen production and hydrogen utilization. The conversion of sugars into pyruvate constitutes the major source of hydrogen, next to the conversion of lactate into acetate. Reactions in the intestines that utilize hydrogen are reducing sulfate to sulfide, converting hydrogen into methane, and converting hydrogen into acetate (80, 81). Some microbial species, mainly belonging to the *Bacillota* (formally Firmicutes) phylum, have been identified as high hydrogen producers, whereas others, such as *Akkermansia muciniphila* and *Faecalibacterium prausnitzii*, produce low amounts of hydrogen (81). Acetogens typically belong to the genera *Ruminococcus, Clostridium*, or *Streptococcus*, whereas only 2 methanogenic species have been isolated from the human colon, i.e., *Methanobrevibacter smithii* and *Methanosphaera stadtmanae* (81, 82). In contrast, sulfur-reducing bacteria are a diverse group of bacteria ubiquitously present in the human intestine (81). Overall, both individual baseline fecal microbiota composition and the capacity of the NDC to selectively stimulate specific species may influence gas production.

##### MRI for symptom evaluation

NDC fermentation results in SCFAs that are osmotically active and attract water to the gut lumen (6, 7). Colonic distention due to accumulation of gas or water may induce abdominal discomfort. MRI allows for small-bowel water quantification, as well as the amount of luminal gas and the colonic volume. So far, the technique has mainly been applied to better understand symptom patterns in patients with irritable bowel syndrome (7, 8).

### Special considerations for children

When assessing gastrointestinal tolerance to NDCs in children, several special considerations not necessarily needed in adults are necessary.

#### Dietary fiber nutritional requirements

Fiber is essential in all children's diets (83). The adequate intake recommendation for dietary fiber for children is based on adult data—an intake of 14 g of dietary fiber per 1000 kcal consumed is recommended (84). Overall, the adequate intakes vary from 19 g/d for children aged 1 to 3 y to 26 and 38 g/d for 14- to 18-year-old girls and boys, respectively (84). The American Academy of Pediatrics and the American Health Foundation also provide fiber intake recommendations for children, making it difficult for clinicians and caregivers to know which guidelines to follow (85). As in adults, children in Western populations have a significant gap between recommended and actual dietary fiber intake (18). The most pronounced gap is in male children (18).

#### Childhood cognitive development

Given the need to report symptoms when assessing gastrointestinal tolerance, the ability of a child to do so relies on cognitive maturity. Several factors affect childhood cognitive development (86). Though it is no longer universally embraced, Piaget's age-based theory is a recognized theoretical framework for describing childhood cognitive development (87). As recognized by Piaget, children pass through 4 cognitive stages. The first (ages 0–2 y) is the sensorimotor stage characterized by the development and usage of the 5 senses and memory development. The second (ages 2–7 y) is the preoperational stage characterized by pretend play and understanding symbols (e.g., letters or numbers) as having meanings. The third stage (ages 7–11 y) is the concrete operational stage in which children begin thinking logically about objects, numbers, categories, and events. Finally, for children ≥12 y, there is the formal operational stage in which the child can think hypothetically (87). Given the cognition and memory required when reporting symptoms, it is not until the latter 2 cognitive stages (i.e., >7 y of age) that children generally can reliably complete inquiries related to gastrointestinal intolerance without a parental or caregiver proxy.

Ultimately, developmentally appropriate and validated measures for children related to gastrointestinal tolerance testing should be used. Examples of such potential measures include the PROMIS Pediatric Pain Interference Scale (88). This scale allows a child 8–17 y of age to self-report symptoms (88). In 2012, a childhood gastrointestinal symptom module known as the PedsQL-Gastrointestinal Symptom and Worry Scale was developed with 76 items and 11 domains (89). Several domains of potential interest for assessing NDC consumption tolerance can be assessed either together or individually, including stomach pain, stomach upset, constipation, diarrhea, and flatulence/bloating (89). The PedsQL Gastrointestinal Symptom and Worry Scale has age-appropriate modules for children as young as 5 y and for parents assessing symptoms of children as young as 2 y; it has been used successfully to differentiate children with a disorder of gut–brain interaction and organic gastrointestinal disorders from healthy children (89, 90). General health-related quality of life can be concomitantly assessed in children using the validated PedsQL Generic Core Scale (91).

Pictorial representations on assessments are believed to offer children more information on how to grade answers on self-reported measures (92). The use of visual representations in pediatric self-report measures can reduce child memory requirements, help maintain attention, and avoid relying solely on verbal and/or reading skills (93). In addition, children's cognitive development can be taken into account by decreasing the number of choices and using developmentally appropriate language (94). Pictorial representations have been used in assessing stool form for several decades, primarily with the Bristol Stool Form Scale, which uses pictorial representations of 7 categories of stool form (71). A modified Bristol Stool Form Scale for children has been developed that decreases the number of categories to 5, and it has been validated in children as young as 8 y and as young as 6 y if the stool form descriptors are read (95, 96). Similarly, though targeted toward adults, an infant stool scale has been developed with pictorial representations of stools in diapers (97).

#### Noninvasive gastrointestinal physiologic assessments

Several techniques are available for the noninvasive assessment of intestinal transit in children. Carmine red, a nonabsorbed material that is easily swallowed, measures whole intestinal transit via determining the time of ingestion to the presentation of red dye in the stool (98). As in adults, breath testing with nonabsorbable substrates such as lactulose can estimate oro-cecal transit time (98). Radio-opaque markers also have been used in children to measure intestinal transit time. To minimize radiation exposure, one approach was to obtain radiographs of the bowel movements themselves rather than the children (99). Finally, a wireless motility capsule has been used successfully to assess transit times in children (100).

#### Prevalence of disorders of gut–brain interaction in children

Like adults, pediatric disorders of gut–brain interaction, including irritable bowel syndrome, are highly prevalent, with up to 25% of children affected worldwide (101, 102). Specific pediatric Rome criteria define these heterogeneous disorders, and some children have abnormal fecal microbiota composition and visceral hypersensitivity (101, 103, 104). Foods with some NDCs, such as fructans, have been shown to exacerbate gastrointestinal symptoms in children with disorders of gut–brain interaction (105, 106).

Information is available regarding gastrointestinal symptoms in the general pediatric population. Though found to be more severe in children who meet the criteria for disorders of gut–brain interaction, abdominal pain is also reported by healthy children when using the abdominal pain index (107). Therefore, similar to assessment in adults, baseline characteristics should be assessed in children before challenging with consumption of an NDC to accurately determine changes in gastrointestinal symptoms.

#### Physiologic differences in children compared with adults

##### Gastrointestinal physiology

Children's gastrointestinal physiology differs from that of adults, which may play a role in NDC tolerance. For example, younger and smaller children (as determined by body surface area) have slower gastric emptying of a standardized solid meal (108). Slower gastric emptying of carbohydrates is associated with improved overall tolerance (109). Also, on average, children aged <3 y have more frequent bowel movements than adults (110). Studies involving children aged >3 y should be designed to account for their higher baseline stool frequency.

##### Intestinal microbiota

The microbiome is an important factor related to the physiologic processing of NDCs once they reach the colon. There are differences between children, adolescents, and adults with respect to both fecal microbiota composition and metabolite production (111). The baseline diet is believed to play a role in determining a child's intestinal microbiota composition (112). Though not demonstrated in otherwise healthy children, fecal microbiota composition is associated with fructan intolerance in children with irritable bowel syndrome (105). Of note, human microbiome-related differences distinguish children with irritable bowel syndrome from healthy children (104).

### Implications

We recognize the importance of developing a scientifically valid approach for assessing tolerance to NDCs. In this Perspective we have provided recommendations for evaluating tolerance of NDCs to a diverse range of stakeholders, including researchers, industry, and regulators. However, other parties, including healthcare providers and consumers, will benefit from better research on NDC tolerance. Indeed, there is a significant fiber gap whereby 90% of women and 97% of men in the United States fail to consume the recommended daily amount of dietary fiber (50). However, this gap could be narrowed by enriching (or fortifying) foods and beverages with dietary fibers (19). As adequate fiber consumption is linked to reduced risk of disease, including cardiovascular disease, type 2 diabetes, and cancer, healthcare providers also have a vested interest in adequate fiber consumption by their patients. The health benefits of adequate consumption of NDCs, including fiber, as well as their functional properties, have contributed to increased NDC use within processed foods and research to develop new NDCs. The guidance provided for conducting trials to assess tolerance to NDCs may also help improve the study replication, thereby improving our understanding of tolerance to NDCs. Ultimately, investigators are responsible for designing and conducting high-quality research trials and adequately reporting the results so that this research area can continue to move forward.

### Conclusions

This report provides recommendations for the research needed to investigate gastrointestinal tolerance to NDCs. The randomized, double-blind, controlled crossover trial approach is recommended for trials on tolerance. Specific recommendations for inclusion and exclusion criteria of study populations, including dietary, health, lifestyle, and medications, also are included. When considering the intervention, fully characterizing the structure, purity, and functionality of the NDC is imperative. NDC delivery via food or beverage should also be considered as responses to NDC within liquid compared with solid formulations can vary. Negative comparators or the control should be formulated to be as similar as possible in appearance, taste, texture, and nutrient content to the test food product but contain no NDC ingredient. When using positive controls, the amount should be similar to the test NDC amount, and the physicochemical properties of the comparator should be considered. As gastrointestinal tolerance refers to the capacity of the body to endure a substance, subjective participant-reported symptoms, assessed via standard gastrointestinal questionnaires, are the primary measures that should be included in tolerance studies. Objective outcome parameters can complement gastrointestinal questionnaires and may be helpful to increase understanding of symptoms. However, these parameters do not measure tolerance, which is symptom based, and therefore, do not replace gastrointestinal questionnaires. Special considerations that should be taken into account when assessing children's gastrointestinal tolerance include their nutritional requirements and cognitive development. The goal is that the recommendations contained within this Perspective will be utilized and that they will aid advances in tolerance research, which ultimately will benefit consumers through the inclusion of acceptable NDCs in food products that narrow the fiber gap.

## Supplementary Material

nmac091_Supplemental_File

## Notes

Opinions expressed in Perspective articles are those of the author(s) and are not attributable to the funder(s) or the sponsor(s) or the publisher, Editor, or Editorial Board of Advances in Nutrition. Individuals with different positions on the topic of a Perspective are invited to submit their comments in the form of a Perspectives article or in a Letter to the Editor.No authors received funding support for this work. Publication fees were covered by the Institute for the Advancement of Food and Nutrition Sciences (IAFNS) Carbohydrate Committee. IAFNS is a nonprofit science organization that pools funding from industry and advances science through the in-kind and financial contributions from private and public sector members.

Author disclosures: HDH has received presentation honoraria from the Beneo, Calorie Control Council, Kellogg's, Nutricia North America, PepsiCo, and Tate & Lyle, received grant support from General Mills and Tate & Lyle, and served on the scientific advisory board of the Grains Food Foundation. BPC receives royalties from the Rome Foundation for the use of the modified Bristol Stool Scale for Children, has received grant funding from the National Institutes of Health (NIDDK DK101688 and DK117219) and intellectual support from NIH DK56338, which funds the Texas Medical Center Digestive Disease Center, and P30 ES030285, which funds the Gulf Coast Center for Precision Environmental Health. WJD has received grant funding from Danstar Ferment AG, Saskatchewan Pulse Growers, Pepsico Inc., and Lallemand Inc. DJL is an independent consultant and works with Biofortis-Merieux Nutrisciences, a contract research organization that conducts clinical studies for food, nutrition, and supplement companies. JLS has current research grants from Taiyo and Barilla in the area of dietary fiber, serves on the Scientific Advisory Board for the Quality Carbohydrate Council, Simply Good Foods, and the Sustainable Nutrition Scientific Board, and is an Academic Advisor for the Carbohydrates Committee for the Institute for the Advancement of Food and Nutrition Sciences (IAFNS). KV has received speaking honoraria from Yakult and Biocodex and received an unrestricted grant from the Société des Produits de Nestlé SA and was a beneficiary of the WK Kellogg chair in Cereal Sciences and Nutrition. All other authors report no conflicts of interest.

Supplemental Figures 1–3 are available from the "Supplementary data" link in the online posting of the article and from the same link in the online table of contents at https://academic.oup.com/advances/.

Abbreviations used: DV, daily value; GSRS, Gastrointestinal Symptom Rating Scale; NDC, nondigestible carbohydrate

SCFA, short-chain fatty acids

## References

[1] Office of Nutrition and Food Labeling, Center for Food Safety and Applied Nutrition, Food and Drug Administration, US Department of Health and Human Services . Science review of isolated and synthetic non-digestible carbohydrates. US Food Drug Adminstration. 2016;1–129.

[2] Holscher HD . Dietary fiber and prebiotics and the gastrointestinal microbiota. Gut Microbes. 2017;8(2):172–84. 28165863 10.1080/19490976.2017.1290756 PMC5390821

[3] U.S. Food and Drug Administration . The declaration of certain isolated or synthetic non-digestible carbohydrates as dietary fiber on nutrition and supplement fact labels; guidance for industry. Washington, DC; 2018.

[4] Food and Drug Administration . Review of the scientific evidence on the physiological effects of certain non-digestible carbohydrates. 2018;1–52.

[5] Franck A . Technological functionality of inulin and oligofructose. Br J Nutr. 2002;87(S2):S287–91. 12088531 10.1079/BJNBJN/2002550

[6] Ruppin H , Bar-Meir S, Soergel KH, Wood CM, Schmitt MG Jr. Absorption of short-chain fatty acids by the colon. Gastroenterology. 1980;78(6):1500–7. 6768637

[7] Major G , Pritchard S, Murray K, Alappadan JP, Hoad CL, Marciani L et al. Colon hypersensitivity to distension, rather than excessive gas production, produces carbohydrate-related symptoms in individuals with irritable bowel syndrome. Gastroenterology. 2017;152(1):124–133.e2. 27746233 10.1053/j.gastro.2016.09.062

[8] Wu J , Masuy I, Biesiekierski JR, Fitzke HE, Parikh C, Schofield L et al. Gut-brain axis dysfunction underlies FODMAP-induced symptom generation in irritable bowel syndrome. Aliment Pharmacol Ther. 2022;55:670–82. 35166384 10.1111/apt.16812

[9] Azpiroz F , Guyonnet D, Donazzolo Y, Gendre D, Tanguy J, Guarner F. Digestive symptoms in healthy people and subjects with irritable bowel syndrome: validation of symptom frequency questionnaire. J Clin Gastroenterol. 2015;49(7):e64–70. 25014236 10.1097/MCG.0000000000000178 PMC4495859

[10] Bonnema AL , Kolberg LW, Thomas W, Slavin JL. Gastrointestinal tolerance of chicory inulin products. J Am Diet Assoc. 2010;110(6):865–8. 20497775 10.1016/j.jada.2010.03.025

[11] Asnicar F , Leeming ER, Dimidi E, Mazidi M, Franks PW, Al Khatib H et al. Blue poo: impact of gut transit time on the gut microbiome using a novel marker. Gut. 2021;70(9):1665–74. 33722860 10.1136/gutjnl-2020-323877 PMC8349893

[12] Cummings JH , Jenkins DJA, Wiggins HS. Measurement of the mean transit time of dietary residue through the human gut. Gut. 1976;17(3):210–8. 1269989 10.1136/gut.17.3.210 PMC1411154

[13] Probert CSJ , Emmett PM, Heaton KW. Some determinants of whole-gut transit time: a population-based study. QJM. 1995;88(5)::311–5. 7796084

[14] Mego M , Accarino A, Tzortzis G, Vulevic J, Gibson G, Guarner F et al. Colonic gas homeostasis: mechanisms of adaptation following HOST-G904 galactooligosaccharide use in humans. Neurogastroenterol Motil. 2017;29(9):1–8. 10.1111/nmo.13080 28418214

[15] Simpson HL , Campbell BJ. Review article: dietary fibre-microbiota interactions. Aliment Pharmacol Ther. 2015;42(2):158–79. 26011307 10.1111/apt.13248 PMC4949558

[16] US Food and Drug Administration . Scientific Evaluation of the Evidence on the Beneficial Physiological Effects of Isolated or Synthetic Non-Digestible Carbohydrates Submitted as a Citizen Petition (21 CFR 10.30): Guidance for Industry. 2018.

[17] Poutanen KS , Fiszman S, Marsaux CFM, Pentikäinen SP, Steinert RE, Mela DJ. Recommendations for characterization and reporting of dietary fibers in nutrition research. Am J Clin Nutr. 2018;108(3):437–44. 29901686 10.1093/ajcn/nqy095 PMC6134289

[18] Clemens R , Kranz S, Mobley AR, Nicklas TA, Raimondi MP, Rodriguez JC et al. Filling America's fiber intake gap: summary of a roundtable to probe realistic solutions with a focus on grain-based foods. J Nutr. 2012;142(7): 1390S–401S. 22649260 10.3945/jn.112.160176

[19] Canene-Adams K , Laurie I, Karnik K, Flynn B, Goodwin W, Pigat S. Estimating the potential public health impact of fibre enrichment: a UK modelling study. Br J Nutr. 2022;1–7. 10.1017/S0007114521004827 PMC9597481 34991735

[20] Food and Drug Administration . Food labeling: revision of the nutrition and supplement facts labels. 81 FR 33741. 2016. 27236870

[21] Brum J , Ramsey D, McRorie J, Bauer B, Kopecky SL. Meta-analysis of usefulness of psyllium fiber as adjuvant antilipid therapy to enhance cholesterol lowering efficacy of statins. Am J Cardiol. 2018;122(7):1169–74. 30078477 10.1016/j.amjcard.2018.06.040

[22] Jovanovski E , Khayyat R, Zurbau A, Komishon A, Mazhar N, Sievenpiper JL et al. Should viscous fiber supplements be considered in diabetes control? Results from a systematic review and meta-analysis of randomized controlled trials. Diabetes Care. 2019;42(5):755–66. 30617143 10.2337/dc18-1126

[23] de Vries J , Birkett A, Hulshof T, Verbeke K, Gibes K. Effects of cereal, fruit and vegetable fibers on human fecal weight and transit time: a comprehensive review of intervention trials. Nutrients. 2016;8(3): 130. 26950143 10.3390/nu8030130 PMC4808860

[24] McRae MP . Effectiveness of fiber supplementation for constipation, weight loss, and supporting gastrointestinal function: a narrative review of meta-analyses. J Chiropractic Med. 2020;19(1):58–64. 10.1016/j.jcm.2019.10.008 PMC7646157 33192192

[25] de Vries J , Le Bourgot C, Calame W, Respondek F. Effects of β-fructans fiber on bowel function: a systematic review and meta-analysis. Nutrients. 2019;11(1):1–17. 10.3390/nu11010091 PMC6356805 30621208

[26] Grabitske HA , Slavin JL. Gastrointestinal effects of low-digestible carbohydrates. Crit Rev Food Sci Nutr. 2009;49(4):327–60. 19234944 10.1080/10408390802067126

[27] Röytiö H , Ouwehand AC. The fermentation of polydextrose in the large intestine and its beneficial effects. Beneficial Microbes. 2014;5(3):305–13. 24736314 10.3920/BM2013.0065

[28] Dahl WJ , Wright AR, Specht GJ, Christman M, Mathews A, Meyer D et al. Consuming foods with added oligofructose improves stool frequency: a randomised trial in healthy young adults. J Nutri Sci. 2014;3:1–8. 10.1017/jns.2014.6 PMC4153283 25191615

[29] Storey D , Lee A, Bornet F, Brouns F. Gastrointestinal responses following acute and medium term intake of retrograded resistant maltodextrins, classified as type 3 resistant starch. Eur J Clin Nutr. 2007;61(11):1262–70. 17299489 10.1038/sj.ejcn.1602642

[30] Goyal RK , Guo Y, Mashimo H. Advances in the physiology of gastric emptying. Neurogastroenterol Motil. 2019;31(4):1–14. 10.1111/nmo.13546 PMC6850045 30740834

[31] Tian S , Sun Y. Influencing factor of resistant starch formation and application in cereal products: a review. Int J Biol Macromol. 2020;149:424–31. 32004604 10.1016/j.ijbiomac.2020.01.264

[32] Weaver CM , Lichtenstein AH, K-E PM, Perspective: Guidelines needed for the conduct of human nutrition randomized controlled trials. Adv Nutr. 2021;12(1):1–3. 33200180 10.1093/advances/nmaa083 PMC7850095

[33] So D , Yao CK, Gill PA, Pillai N, Gibson PR, Muir JG. Screening dietary fibres for fermentation characteristics and metabolic profiles using a rapid in vitro approach: implications for irritable bowel syndrome. Br J Nutr. 2021;126(2):208–18. 33028442 10.1017/S0007114520003943

[34] Wolever TMS , Meynier A, Jenkins AL, Brand-Miller JC, Atkinson FS, Gendre D et al. Glycemic index and insulinemic index of foods: an interlaboratory study using the ISO 2010 method. Nutrients. 2019;11(9):1–12. 10.3390/nu11092218 PMC6770275 31540317

[35] Ford AL , Nagulesapillai V, Piano A, Auger J, Girard SA, Christman M et al. Microbiota stability and gastrointestinal tolerance in response to a high-protein diet with and without a prebiotic, probiotic, and synbiotic: a randomized, double-blind, placebo-controlled trial in older women. J Acad Nutr Diet. 2020;120(4):500–16.e10. 32199523 10.1016/j.jand.2019.12.009

[36] Meance S , Cayuela C, Raimondi A, Turchet P, Lucas C, Antoine JM. Recent advances in the use of functional foods: effects of the commercial fermented milk with Bifidobacterium Animalis strain DN-173 010 and yoghurt strains on gut transit time in the elderly. Microb Ecol Health Dis. 2003;15:15–22.

[37] Morelli L , Pellegrino P. A critical evaluation of the factors affecting the survival and persistence of beneficial bacteria in healthy adults. Beneficial Microbes. 2021;12(4):321–31. 10.3920/BM2021.0017 34323162

[38] Johnson AJ , Zheng JJ, Kang JW, Saboe A, Knights D, Zivkovic AM. A guide to diet-microbiome study design. Front Nutr. 2020;7:1–16. 32596250 10.3389/fnut.2020.00079 PMC7303276

[39] Centers for Disease Control and Prevention . Chronic Disease in America. 2020.

[40] Schmulson MJ , Drossman DA. What is new in Rome IV. J Neurogastroenterol Motil. 2017;23(2):151–63. 28274109 10.5056/jnm16214 PMC5383110

[41] Thapar N , Benninga MA, Crowell MD, Di Lorenzo C, Mack I, Nurko S et al. Paediatric functional abdominal pain disorders. Nat Rev Dis Primers. 2020;6(1):89. 33154368 10.1038/s41572-020-00222-5

[42] Black CJ , Drossman DA, Talley NJ, Ruddy J, Ford AC. Functional gastrointestinal disorders: advances in understanding and management. Lancet North Am Ed. 2020;396(10263):1664–74. 10.1016/S0140-6736(20)32115-2 33049221

[43] Black CJ , Ford AC. Global burden of irritable bowel syndrome: trends, predictions and risk factors. Nat Rev Gastroenterol Hepatol. 2020;17(8):473–86. 32296140 10.1038/s41575-020-0286-8

[44] US Census Bureau . 65 and older population grows rapidly as baby bommers age. 2020. Available from: https://www.census.gov/newsroom/press-releases/2020/65-older-population-grows.html

[45] Dumic I , Nordin T, Jecmenica M, Stojkovic Lalosevic M, Milosavljevic T, Milovanovic T. Gastrointestinal tract disorders in older age. Can J Gastroenterol Hepatol. 2019;2019:6757524. 30792972 10.1155/2019/6757524 PMC6354172

[46] Espinosa De Ycaza AE , Donegan D, Jensen MD. Long-term metabolic risk for the metabolically healthy overweight/obese phenotype. Int J Obes. 2018;42(3):302–9. 10.1038/ijo.2017.233 PMC5867190 29064474

[47] Meleine M , Matricon J. Gender-related differences in irritable bowel syndrome: potential mechanisms of sex hormones. World J Gastroenterol. 2014;20(22):6725–43. 24944465 10.3748/wjg.v20.i22.6725 PMC4051914

[48] Prusator DK , Chang L. Sex-related differences in GI disorders. Handb Exp Pharmacol. 2017;239:177–92. 28233176 10.1007/164_2016_121

[49] Zia JK , Heitkemper MM. Upper gastrointestinal tract motility disorders in women, gastroparesis, and gastroesophageal reflux disease. Gastroenterol Clin North Am. 2016;45(2):239–51. 27261896 10.1016/j.gtc.2016.02.003

[50] U.S. Department of Agriculture and U.S. Department of Health and Human Services . Dietary Guidelines for Americans, 2020–2025. 9th Ed. 2020. Available from: DietaryGuidelines.gov.

[51] Papoutsopoulou S , Satsangi J, Campbell BJ, Probert CS. Review article: impact of cigarette smoking on intestinal inflammation—direct and indirect mechanisms. Aliment Pharmacol Ther. 2020;51(12):1268–85. 32372449 10.1111/apt.15774

[52] Lohse T , Rohrmann S, Bopp M, Faeh D. Heavy smoking is more strongly associated with general unhealthy lifestyle than obesity and underweight. PLoS One. 2016;11(2):1–13. 10.1371/journal.pone.0148563 PMC4765891 26910775

[53] Philpott HL , Nandurkar S, Lubel J, Gibson PR. Drug-induced gastrointestinal disorders. Postgrad Med J. 2014;90(1065):411–9. 24942356 10.1136/postgradmedj-2013-100316rep

[54] McFarland LV . Epidemiology, risk factors and treatments for antibiotic-associated diarrhea. Dig Dis. 1998;16(5):292–307. 9892789 10.1159/000016879

[55] Wiström J , Norrby SR, Myhre EB, Eriksson S, Granström G, Lagergren L et al. Frequency of antibiotic-associated diarrhoea in 2462 antibiotic-treated hospitalized patients: a prospective study. J Antimicrob Chemother. 2001;47(1):43–50. 11152430 10.1093/jac/47.1.43

[56] Marco ML , Sanders ME, Gänzle M, Arrieta MC, Cotter PD, De Vuyst L et al. The international scientific association for probiotics and prebiotics (ISAPP) consensus statement on fermented foods. Nat Rev Gastroenterol Hepatol. 2021;18(3):196–208. 33398112 10.1038/s41575-020-00390-5 PMC7925329

[57] Maki KC , Rains TM, Kelley KM, Cook CM, Schild AL, Gietl E. Fibermalt is well tolerated in healthy men and women at intakes up to 60 g/d: a randomized, double-blind, crossover trial. Int J Food Sci Nutr. 2013;64(3):274–81. 23110312 10.3109/09637486.2012.738652

[58] Iriondo-Dehond M , Iriondo-Dehond A, Herrera T, Fernandez-Ferdandez AM, Zorzano COS, Miguel E et al. Sensory acceptance, appetite control and gastrointestinal tolerance of yogurts containing coffee-cascara extract and inulin. Nutrients. 2020; 12(3):627. 32121016 10.3390/nu12030627 PMC7146162

[59] Crincoli CM , Garcia-Campayo V, Rihner MO, Nikiforov AI, Liska DA, van de Ligt JLG. Evaluation of the gastrointestinal tolerability of corn starch fiber, a novel dietary fiber, in two independent randomized, double-blind, crossover studies in healthy men and women. Int J Food Sci Nutr. 2016;67(7):844–56. 27346078 10.1080/09637486.2016.1198891

[60] Holscher HD , Doligale JL, Bauer LL, Gourineni V, Pelkman CL, Fahey GC et al. Gastrointestinal tolerance and utilization of agave inulin by healthy adults. Food Funct. 2014;5(6):1142–9. 24664349 10.1039/c3fo60666j

[61] Deehan EC , Yang C, Perez-Muñoz ME, Nguyen NK, Cheng CC, Triador L et al. Precision microbiome modulation with discrete dietary fiber structures directs short-chain fatty acid production. Cell Host Microbe. 2020;27(3):389–404.e6. 32004499 10.1016/j.chom.2020.01.006

[62] Dimenäs E , Glise H, Hallerbäck B, Hernqvist H, Svedlund J, Wiklund I. Quality of life in patients with upper gastrointestinal symptoms: an improved evaluation of treatment regimens?. Scand J Gastroenterol. 1993;28(8):681–7. 8210982 10.3109/00365529309098272

[63] Svedlund J , Sjödin I, Dotevall G. GSRS-A clinical rating scale for gastrointestinal symptoms in patients with irritable bowel syndrome and peptic ulcer disease. Dig Dis Sci. 1988;33(2):129–34. 3123181 10.1007/BF01535722

[64] Dimenäs E , Carlsson G, Glise H, Israelsson B, Wiklund I. Relevance of norm values as part of the documentation of quality of life instruments for use in upper gastrointestinal disease. Scand J Gastroenterol. 1996;31(sup221):8–13. 10.3109/00365529609095544 9110389

[65] Dennis-Wall JC , Burns AM, Solch RJ, Ukhanova M, Dahl WJ, Christman MC et al. A beverage containing orange pomace improves laxation and modulates the microbiome in healthy adults: a randomised, blinded, controlled trial. J Funct Foods. 2019;60:103438.

[66] Alyousif Z , Mendoza DR, Auger J, De Carvalho V, Amos S, Sims C et al. Gastrointestinal tolerance and microbiome response to snacks fortified with pea hull fiber: a randomized trial in older adults. Curr Dev Nutr. 2020;4(2):1–11. 32025615 10.1093/cdn/nzaa005 PMC6994441

[67] Hughes C , Davoodi-Semiromi Y, Colee JC, Culpepper T, Dahl WJ, Mai V et al. Galactooligosaccharide supplementation reduces stress-induced gastrointestinal dysfunction and days of cold or flu: a randomized, double-blind, controlled trial in healthy university students. Am J Clin Nutr. 2011;93(6):1305–11. 21525194 10.3945/ajcn.111.014126

[68] Wilder-Smith CH , Olesen SS, Materna A, Drewes AM. Fermentable sugar ingestion, gas production, and gastrointestinal and central nervous system symptoms in patients with functional disorders. Gastroenterology. 2018;155(4):1034–1044.e6. 30009815 10.1053/j.gastro.2018.07.013

[69] Bovenschen HJ , Janssen MJR, Van Oijen MGH, Laheij RJF, Van Rossum LGM, Jansen J. Evaluation of a gastrointestinal symptoms questionnaire. Dig Dis Sci. 2006;51(9):1509–15. 16927133 10.1007/s10620-006-9120-6

[70] Heaton KW , Ghosh S, Braddon FEM. How bad are the symptoms and bowel dysfunction of patients with the irritable bowel syndrome? A prospective, controlled study with emphasis on stool form. Gut. 1991;32(1):73–9. 1991641 10.1136/gut.32.1.73 PMC1379218

[71] Lewis SJ , Heaton KW. Stool form scale as a useful guide to intestinal transit time. Scand J Gastroenterol. 1997;32(9):920–4. 9299672 10.3109/00365529709011203

[72] Riegler G , Esposito I. Bristol scale stool form. A still valid help in medical practice and clinical research. Tech Coloproctol. 2001;5(3):163–4. 11875684 10.1007/s101510100019

[73] Blake MR , Raker JM, Whelan K. Validity and reliability of the Bristol Stool Form Scale in healthy adults and patients with diarrhoea-predominant irritable bowel syndrome. Aliment Pharmacol Ther. 2016;44(7):693–703. 27492648 10.1111/apt.13746

[74] Levitt MD . Production and excretion of hydrogen gas in man. N Engl J Med. 1969;281:122–7. 5790483 10.1056/NEJM196907172810303

[75] Christl S , Murgatroyd P, Gibson GR, Cummings JH. Production, metabolism, and excretion of hydrogen in the large intestine. Gastroenterology. 1992;102(4):1269–77. 1551534

[76] Houben E , de Preter V, Billen J, van Ranst M, Verbeke K. Additional value of CH4 measurement in a combined 13C/H2 lactose malabsorption breath test: a retrospective analysis. Nutrients. 2015;7(9):7469–85. 26371034 10.3390/nu7095348 PMC4586543

[77] van der Sijp J , Kamm MA, Nightingale JMD, Britton KE, Mather SJ, Morris GP et al. Radioisotope determination of regional colonic transit in severe constipation: comparison with radio opaque markers. Gut. 1993;34(3):402–8. 8472991 10.1136/gut.34.3.402 PMC1374150

[78] Cummings JH , Wiggins HS. Transit through the gut measured by analysis of a single stool. Gut. 1976;17(3):219–23. 1269990 10.1136/gut.17.3.219 PMC1411155

[79] Baer DJ , Gebauer SK, Novotny JA. Walnuts consumed by healthy adults provide less available energy than predicted by the Atwater Factors. J Nutr. 2015;9–13. 26581681 10.3945/jn.115.217372

[80] Smith NW , Shorten PR, Altermann EH, Roy NC, McNabb WC. Hydrogen cross-feeders of the human gastrointestinal tract. Gut Microbes. 2019;10(3):270–88. 30563420 10.1080/19490976.2018.1546522 PMC6546324

[81] Carbonero F , Benefiel AC, Gaskins HR. Contributions of the microbial hydrogen economy to colonic homeostasis. Nat Rev Gastroenterol Hepatol. 2012;9(9):504–18. 22585131 10.1038/nrgastro.2012.85

[82] Nava GM , Carbonero F, Croix JA, Greenberg E, Gaskins HR. Abundance and diversity of mucosa-associated hydrogenotrophic microbes in the healthy human colon. ISME J. 2012;6(1):57–70. 21753800 10.1038/ismej.2011.90 PMC3246246

[83] Hojsak I , Benninga MA, Hauser B, Kansu A, Kelly VB, Stephen AM et al. Benefits of dietary fibre for children in health and disease. Arch Dis Child. 2022;107(11):973–9. 35277379 10.1136/archdischild-2021-323571 PMC9606532

[84] Trumbo P , Schlicker S, Yates AA, Poos M. Dietary reference intakes for energy, carbohydrate, fiber, fat, fatty acids, cholesterol, protein and amino acids.J Am Diet Assoc. 102:1621–30. 10.1016/s0002-8223(02)90346-9 12449285

[85] Korczak R , Kamil A, Fleige L, Donovan SM, Slavin JL. Dietary fiber and digestive health in children. Nutr Rev. 2017;75(4):241–59. 28586481 10.1093/nutrit/nuw068

[86] Guinosso SA , Johnson SB, Riley AW. Multiple adverse experiences and child cognitive development. Pediatr Res. 2016;79(1–2):220–6. 26460522 10.1038/pr.2015.195

[87] HK S , M C. StatsPearls. Piaget. Treasure Island, FL; 2020.

[88] Varni JW , Stucky BD, Thissen D, Dewitt EM, Irwin DE, Lai J-S et al. PROMIS pediatric pain interference scale: an item response theory analysis of the pediatric pain item bank. J Pain. 2010;11(11):1109–19. 20627819 10.1016/j.jpain.2010.02.005 PMC3129595

[89] Varni JW , Kay MT, Limbers CA, Franciosi JP, Pohl JF. PedsQL gastrointestinal symptoms module item development: qualitative methods. Journal of Pediatric Gastroenterol Nutri. 2012;54(5):664–71. 10.1097/MPG.0b013e31823c9b88 22008958

[90] Varni JW , Bendo CB, Shulman RJ, Self MM, Nurko S, Franciosi JP et al. Interpretability of the PedsQL^TM^ gastrointestinal symptoms scales and gastrointestinal worry scales in pediatric patients with functional and organic gastrointestinal diseases. J Pediatr Psychol. 2015;40(6):591–601. 25682210 10.1093/jpepsy/jsv005 PMC4469917

[91] Varni S M , Kurtin P. PedsQL^TM^ 4 .0 : reliability and validity of the pediatric quality of life inventory^TM^ Version 4.0 generic core scales in healthy and patient populations med care. 2001;39:800–12. 10.1097/00005650-200108000-00006 11468499

[92] Cremeens J , Eiser C, Blades M. Characteristics of health-related self-report measures for children aged three to eight years: a review of the literature. Qual Life Res. 2006;15(4):739–54. 16688506 10.1007/s11136-005-4184-x

[93] Salmon K , Yao J, Berntsen O, Pipe ME. Does providing props during preparation help children to remember a novel event?. J Exp Child Psychol. 2007;97(2):99–116. 17328907 10.1016/j.jecp.2007.01.001

[94] Varni JW , Waldron SA, Gragg RA, Rapoff MA, Bernstein BH, Lindsley CB et al. Development of the Waldron/Varni Pediatric Pain Coping Inventory. Pain. 1996;67(1):141–50. 8895242 10.1016/0304-3959(96)03077-1

[95] Chumpitazi BP , Lane MM, Czyzewski DI, Weidler EM, Swank PR, Shulman RJ. Creation and initial evaluation of a stool form scale for children. J Pediatr. 2010;157(4):594–7. 20826285 10.1016/j.jpeds.2010.04.040 PMC2937014

[96] Lane MM , Czyzewski DI, Chumpitazi BP, Shulman RJ. Reliability and validity of a modified bristol stool form scale for children. J Pediatr. 2011;159(3):437–441.e1. 21489557 10.1016/j.jpeds.2011.03.002 PMC3741451

[97] Bekkali N , Hamers SL, Reitsma JB, Van Toledo L, Benninga MA. Infant stool form scale: development and results. J Pediatr. 2009;154(4): 521–6.e1. 19054528 10.1016/j.jpeds.2008.10.010

[98] Chumpitazi BP , Weidler EM, Shulman RJ. Lactulose breath test gas production in childhood IBS is associated with intestinal transit and boewl movement frequency. J Pediatr Gastroenterol Nutr. 2017;64(4):541–5. 27276436 10.1097/MPG.0000000000001295 PMC5145773

[99] Corazziari E , Cucchiara S, Staiano A, Romaniello G, Tamburrini O, Torsoli A et al. Gastrointestinal transit time, frequency of defecation, and anorectal manometry in healthy and constipated children. J Pediatr. 1985;106(3):379–82. 3973774 10.1016/s0022-3476(85)80660-0

[100] Shulman RJ , Chumpitazi BP, Abdel-Rahman SM, Garg U, Musaad S, Kearns GL. Randomised trial: peppermint oil (menthol) pharmacokinetics in children and effects on gut motility in children with functional abdominal pain. Br J Clin Pharmacol. 2022;88(3):1321–33. 34528282 10.1111/bcp.15076 PMC8863319

[101] Chumpitazi BP , Shulman RJ. Underlying molecular and cellular mechanisms in childhood irritable bowel syndrome. Mol Cell Pediatr. 2016;3:11. 26883355 10.1186/s40348-016-0036-8 PMC4755958

[102] Robin SG , Keller C, Zwiener R, Hyman PE, Nurko S, Saps M et al. Prevalence of pediatric functional gastrointestinal disorders utilizing the Rome IV criteria. J Pediatr. 2018;195:134–9. 29398057 10.1016/j.jpeds.2017.12.012

[103] Hyams JS , Di Lorenzo C, Saps M, Shulman RJ, Staiano A, Van Tilburg M. Childhood functional gastrointestinal disorders: child/adolescent. Gastroenterology. 2016;150(6):1456–68.e2.

[104] Hollister EB , Oezguen N, Chumpitazi BP, Luna RA, Weidler EM, Rubio-Gonzales M et al. Leveraging human microbiome features to diagnose and stratify children with irritable bowel syndrome. J Mol Diagn. 2019;21(3):449–61. 31005411 10.1016/j.jmoldx.2019.01.006 PMC6504675

[105] Chumpitazi BP , Hoffman KL, Smith DP, McMeans AR, Musaad S, Versalovic J et al. Fructan-sensitive children with irritable bowel syndrome have distinct gut microbiome signatures. Aliment Pharmacol Ther. 2021;53:499–509. 33314183 10.1111/apt.16204 PMC8281336

[106] Chumpitazi BP , Weidler EM, Lu DY, Tsai CM, Shulman RJ. Self-perceived food intolerances are common and associated with clinical severity in childhood irritable bowel syndrome. J Acad Nutr Dietetics. 2016;116(9):1458–64. 10.1016/j.jand.2016.04.017 PMC5003668 27316779

[107] Laird KT , Sherman AL, Smith CA, Walker LS. Validation of the abdominal pain index using a revised scoring method. J Pediatr Psychol. 2015;40(5):517–25. 25617048 10.1093/jpepsy/jsu118 PMC4502391

[108] Wong GK , Shulman RJ, Chumpitazi BP. Gastric emptying scintigraphy results in children are affected by age, anthropometric factors, and study duration. Neurogastroenterol Motil. 2015;27(3):356–62. 25557417 10.1111/nmo.12499 PMC4339628

[109] Chumpitazi BP . Update on dietary management of childhood functional abdominal pain disorders. Gastroenterol Clin North Am. 2018;47(4):715–26. 30337028 10.1016/j.gtc.2018.07.001 PMC6476188

[110] Baker SS , Liptak GS, Colletti RB, Croffie JM, De Lorenzo C, Ector W et al. Constipation in infants and children: evaluation and treatment. J Pediatr Gastroenterol Nutr. 1999;29(5):612–26. 10554136 10.1097/00005176-199911000-00029

[111] Hollister EB , Riehle K, Luna RA, Weidler EM, Rubio-Gonzales M, Mistretta TA et al. Structure and function of the healthy pre-adolescent pediatric gut microbiome. Microbiome. 2015;3(1):36. 26306392 10.1186/s40168-015-0101-x PMC4550057

[112] De Filippo C , Cavalieri D, Di Paola M, Ramazzotti M, Poullet JB, Massart S et al. Impact of diet in shaping gut microbiota revealed by a comparative study in children from Europe and rural Africa. Proc Natl Acad Sci. 2010;107(33):14691–6. 20679230 10.1073/pnas.1005963107 PMC2930426

