# Supplementary material for: Perspective: Assessing Tolerance to Nondigestible Carbohydrate Consumption
Source: Adv Nutr. 2022 Aug 30;13(6):2084–97. doi: 10.1093/advances/nmac091 (PMC9776727; doi:10.1093/advances/nmac091)
Supplement: nmac091_Supplemental_File [file nmac091_supplemental_file.pdf]

Online Supplemental Material
Perspective: Assessing tolerance to non-digestible carbohydrate consumption
Holscher HD, et al.

Supplemental Figure 1: Daily Record Example (1,2)

BOWEL FUNCTION AND GASTROINTESTINAL TOLERANCE FACTORS
DAY OF WEEK: __________ DATE: _________

Over the past 24 hours, please indicate the severity of the following factors:

	1 (Absent)	2 (Mild)	3 (Moderate)	4 (Severe)
**Burping**				
**Cramping/pain**				
**Distension/bloating**				
**Flatulence/gas**				
**Nausea**				
**Reflux (heartburn)**				
**Rumblings**				

Did you have any bowel movements today? ☐ Yes ☐ No

(If Yes, please complete the following):

Time (AM or PM)

Consistency*						
1	2	3	4	5	6	7

Ease of Passage**				
1	2	3	4	5

A consistency score and ease of passage score should be recorded for each bowel movement.

Stool Consistency Ratings (see example image in the Bristol Stool Scale)*
1 = Separate hard lumps, like nuts
2 = Sausage-shaped but lumpy
3 = Like a sausage but with cracks on surface
4 = Like a sausage or snake, smooth and soft
5 = Soft blobs with clear-cut edges
6 = Fluffy pieces with ragged edges, mushy
7 = Watery, no solid pieces, entirely liquid

Ease of Passage Ratings**

1 = Very easy
2 = Easy
3 = Neither easy nor difficult
4 = Difficult
5 = Very difficult

Supplemental Figure 2: Bristol Stool Chart (3,4)

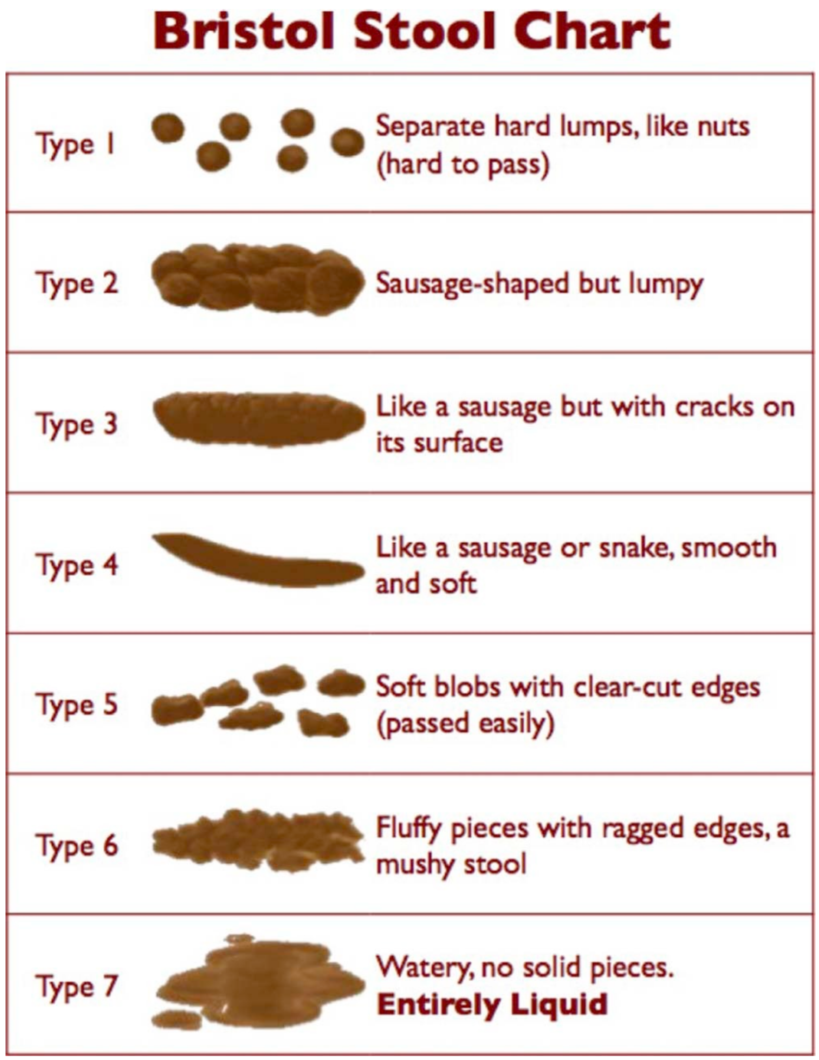

Reprinted with permission from Saad RJ, Rao SS, Koch KL, Kuo B, Parkman HP, McCallum RW, et al. Do stool form and frequency correlate with whole-gut and colonic transit? Results from a multicenter study in constipated individuals and healthy controls. Am J Gastroenterol 2010;105:403-1 (3), and adapted from Lewis SJ, Heaton KW (4).

Supplemental Figure 3. Weekly Tolerance Questionnaire Example (1,5,6)

Gastrointestinal Tolerability Questionnaire

Subject Initials ___ ___ ___ Subject No. _______

Visit No._______ Date ______________

Question	Answer		
1) Did you experience any nausea in the past 7 days?	☐ Yes ☐ No<br><br>If yes, how would you rate the amount of nausea?		
	No more than usual ☐	Somewhat more than usual ☐	Much more than usual ☐
2) Did you experience any bloating in past 7 days?	☐ Yes ☐ No<br><br>If yes, how would you rate the amount of bloating?		
	No more than usual ☐	Somewhat more than usual ☐	Much more than usual ☐
3) Did you experience any gastrointestinal rumblings in the past 7 days?	☐ Yes ☐ No<br><br>If yes, how would you rate the amount of gastrointestinal rumblings?		
	No more than usual ☐	Somewhat more than usual ☐	Much more than usual ☐

Subject's Initials ___________ Date ______________

4) Did you experience any gas/flatulence in the past 7 days?	☐ Yes ☐ No<br>If yes, how would you rate the amount of gas/flatulence?		
	No more than usual	Somewhat more than usual	Much more than usual
	☐	☐	☐
5) Did you experience any abdominal pain in the past 7 days?	☐ Yes ☐ No<br>If yes, how would you rate the amount of abdominal pain?		
	No more than usual	Somewhat more than usual	Much more than usual
	☐	☐	☐
6) Did you experience any diarrhea (watery stools) in the past 7 days?	☐ Yes ☐ No<br>If yes, how would you rate the amount of diarrhea (watery stools)?		
	No more than usual	Somewhat more than usual	Much more than usual
	☐	☐	☐

Coding: no more than usual = 0, somewhat more than usual = 1, much more than usual = 2

Supplemental References:

1. Holscher HD, Doligale JL, Bauer LL, Gourineni V, Pelkman CL, Fahey GC, Swanson KS. Gastrointestinal tolerance and utilization of agave inulin by healthy adults. Food Function 2014;1142–9.
2. Vester Boler BM, Rossoni Serao MC, Bauer LL, Staeger MA, Boileau TW, Swanson KS, Fahey GC, Flood MT, Auerbach MH, Craig SAS, et al. Digestive physiological outcomes related to polydextrose and soluble maize fibre consumption by healthy adult men. Br J Nutr. 2011;106:1864–71.
3. Saad RJ, Rao SSC, Koch KL, Kuo B, Parkman HP, McCallum RW, Sitrin MD, Wilding GE, Semler JR, Chey WD. Do stool form and frequency correlate with whole-gut and colonic transit; Results from a multicenter study in constipated individuals and healthy controls. Am J Gastroenterol. 2009;105:403–11.
4. Lewis SJ, Heaton KW. Stool form scale as a useful guide to intestinal transit time. Scand J Gastroenterol. 1997;32:920–4.
5. Maki KC, Rains TM, Kelley KM, Cook CM, Schild AL, Gietl E. Fibermalt is well tolerated in healthy men and women at intakes up to 60 g/d: a randomized, double-blind, crossover trial. Int J Food Sci Nutr. 2013;64:274–81.
6. Deehan EC, Yang C, Perez-Muñoz ME, Nguyen NK, Cheng CC, Triador L, Zhang Z, Bakal JA, Walter J. Precision microbiome modulation with discrete dietary fiber structures directs short-chain fatty acid production. Cell Host Microbe. 2020;27:389-404.e6.
